# Noncommunicable disease risk factors and cardiovascular risk among adults in Ethiopia: A cross‐sectional study

**DOI:** 10.1002/puh2.133

**Published:** 2023-10-21

**Authors:** Tilahun Tewabe Alamnia, Ginny M. Sargent, Matthew Kelly

**Affiliations:** ^1^ Department of Global Health National Centre for Epidemiology and Population Health the Australian National University Canberra Australia; ^2^ Department of Health Sciences College of Medical and Health Sciences Bahir Dar University Bahir Dar Ethiopia; ^3^ PHxchange National Centre for Epidemiology and Population Health the Australian National University Canberra Australia

**Keywords:** chronic disease predictors, epidemiology, Ethiopia, population health

## Abstract

**Background:**

Noncommunicable diseases (NCDs) are rising in developing countries, posing substantial health, social, and economic consequences. Targeted action to address NCDs is difficult without data on the prevalence and social distribution of the risk factors.

**Methods:**

A cross‐sectional survey was conducted among randomly sampled adults in Bahir Dar, Northwest Ethiopia. Sociodemographic, lifestyle risk factors, knowledge, and anthropometric measurements were collected. Chi‐square and logistic regression analyses were conducted to identify predictors for NCD risk factors and cardiovascular risk.

**Results:**

This study involved 417 participants, with a mean age of 35.6 ± 12.6 years, in which 54.7% were females. The prevalence of inadequate fruit/vegetable intake was 95.7%, alcohol consumption 53.0%, physical inactivity 37.9%, tobacco use 1.9%, khat use 5.1%, hypertension 20.7%, overweight/obesity 29.6%, and abdominal obesity 67.4%. Overall, 55% of adults have clusters of three or more risk factors, and 14.6% have a moderate (10%–20%) 10‐year cardiovascular risk. Factors associated with NCD risk factors are sex, age, religion, marital status, occupation, and income. Ten‐year cardiovascular risk is higher in men (adjusted odd ratio (OR) 4.2, 95% CI 1.5–11.4), in adults with abdominal obesity (adjusted OR 5.8, 95% CI 1.4–24.2), and in those with clustered risk factors (adjusted OR 4.0, 95% CI 1.6–10.3).

**Conclusion:**

This study shows that adults in Northwest Ethiopia are at high risk of developing NCDs. Coordinated, multisectoral interventions at all levels of the socio‐ecological model are needed to reduce the risk factors.

## Graphical abstract

This community‐based survey showed that noncommunicable diseases risk factors are widespread in the study population: Inadequate fruit/vegetable intake is 96%, alcohol consumption 53%, physical inactivity 38%, and tobacco use 2%; 55% of adults have clusters of three or more risk factors, and 15% have a moderate level 10‐year cardiovascular risk.

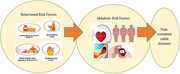

## INTRODUCTION

Noncommunicable diseases (NCDs) are the leading cause of death worldwide. The highest burden is in low‐ and middle‐income countries, where more than three‐quarters (77%) of NCD‐related deaths and 86% of NCD‐related premature deaths occur [1]. The NCDs that are responsible for the majority of the deaths are cardiovascular diseases (CVDs) (46%), cancers (22%), chronic obstructive pulmonary diseases (11%), and diabetes (4%) [1]. Multiple complex and interacting socioeconomic and environmental factors such as globalization, rapid urbanization, sedentary work, unhealthy diet, and population aging aggravate the prevalence of NCDs [2, 3, 4].

NCDs impact countries’ health and social and economic sectors [5] and hinder the progress toward the 2030 Sustainable Development Goals (SDGs). SDG 3.4 targets reducing premature mortality from NCDs by one third by 2030 through prevention, treatment, and promotion of mental health and well‐being [6]. The World Health Organization (WHO) has also proposed targets to reduce NCD risk factors by 2025, such as reducing alcohol use and physical inactivity by 10%, high salt intake and tobacco use by 30%, and hypertension by 25% [7].

Ethiopia has undergone several changes in the past few decades: rapid urbanization, improved education access, maternal and child health services, the health extension program, and healthcare access, as well as overall poverty reduction [8]. Ethiopia is in a stage of mid‐epidemiological transition, with a double burden of both chronic diseases and infectious diseases [9, 10]. The country has an estimated population of 120 million [11], with more than half of the population in the adult age group and demographic transition underway [12].

In 2019, it was estimated that 43% of deaths in Ethiopia were due to NCDs, with CVDs, the most common NCDs, responsible for more than 16% of the deaths [13]. The government has developed a national guideline for NCD management [14]; however, the main focus of the country's health system is still on maternal and child health programs and infectious disease control. Inadequate numbers of trained staff, lack of guidelines and protocols, low service availability, shortages of resources, and competition for health resources are identified gaps to tackle NCDs in the population [15].

Effective policy action requires evidence regarding the prevalence of NCD risk factors and people's knowledge about these risks. One national NCD risk factor survey of 9800 people aged 15–69 years was conducted in 2015 [16]. However, due to issues with sample size and scope, this survey did not provide the regional/district level distribution of risk factors, nor evidence about people's knowledge about the risk factors. Some regional studies in Ethiopia, such as in Mekelle [17] and Southwest Ethiopia [18], have provided some regional insights about risk factors. However, their results are not generalizable to various contexts in the country, such as cultures, traditions, taboos, and ways of life.

This study describes the prevalence of NCD risk factors among adults in Bahir Dar, the clustering of risk factors, and CVD risk levels in the community.

## METHODS

### Study design and setting

This community‐based quantitative cross‐sectional study was conducted between 10 May 2021 and 20 June 2021 and is reported in‐line with the Strengthening the Reporting of Observational Studies in Epidemiology (STROBE) statement [19]. This study was conducted in Bahir Dar, Northwest Ethiopia. Bahir Dar is the main town of Amhara regional state, located 565 km from Addis Ababa, Ethiopia, with an estimated population of 400,000. The town comprises six sub‐cities: *Belay Zeleke, Tana, Atse Minilk, Atse Tewodros, Fasilo, and Shumabo*, and 17 administrative units called *kebele* in the local Amharic language. Bahir Dar has one referral hospital, two district‐level public hospitals, and ten primary healthcare centers, mainly providing curative health services to the population. Shortages of equipment and supplies, lack of resources and trained manpower, and poor health insurance coverage remain challenges to providing appropriate healthcare services, including NCD prevention.

### Sample size and sampling techniques

The calculated sample size for this study was 423. We employed a systematic random sampling technique to select study participants from households in Bahir Dar. The number of households in each sub‐city was obtained from administrative offices. Data collectors randomly started at a household in each sub‐city and moved through a predetermined interval (every 28 households) to locate the next study household, which was obtained by dividing the estimated number of households (*N* = 12,000) by the target sample size (*n* = 423).

Adults between the ages of 18 and 65 years who have lived in Bahir Dar for at least 6 months and those who were able to provide the necessary information were eligible to participate. In households with more than one eligible participant, one was selected using a lottery method. If there was no eligible participant, the household was skipped, and recruitment continued at the adjacent household. If an eligible participant in the household was unavailable at the time of data collection, a revisit was arranged.

### Study variables, instruments, and data collection

Four health professionals (nurses) were trained for two days for data collection. Face‐to‐face interviews were conducted using a structured questionnaire. The questionnaire was prepared by adopting and modifying the WHO STEPS tool [20], and similar studies [21, 22]. It consists of four major parts: sociodemographic characteristics, lifestyle risk factors (dietary habit, physical activity, alcohol consumption, tobacco use, and *khat* chewing), knowledge regarding NCDs, and physical measurements. The questionnaire was prepared in English and translated into *Amharic* (the local language) for data collection. The questionnaire was pretested in a 5% of the sample size to assess participants’ responses and clarity of the questions.

Physical measurements, including weight, height, waist/hip circumference, and blood pressure (BP), were collected using standardized measuring tools. BP measurements were performed twice; however, if the difference between two systolic BP values was above 20 mmHg, a third measurement was performed. Table 1 outlines the classification of measurement variables.

**TABLE 1:** Operational definitions for the study of noncommunicable disease risk factors in Bahir Dar, Northwest Ethiopia.

Outcome variables	Classification of variable
Adequate fruit and vegetable intake	The consumption of at least five servings of fruit and vegetables per day [23]
Sufficient physical activity	A minimum of 150 min of moderate‐intensity or 75 min of vigorous‐intensity activity per week [24]
Alcohol consumption	Former alcohol consumer (have consumed alcohol products in the past but not currently), current alcohol consumer have consumed alcohol within the past 30 days), and nonconsumer (never consumed alcoholic beverages) [25]
Tobacco use	Former tobacco users (have used tobacco products in the past but not currently), current tobacco users (have used tobacco products within the past 30 days), and nonsmokers (never used tobacco products) [26, 27]
Khat chewing	Former *khat* chewers (have chewed *khat* in the past but not currently), current *khat* chewers (have chewed *khat* within the past 30 days), and non‐chewers (never chewed *khat*) [25, 27]
Overweight and obesity	Overweight: BMI between 25 and 29.9 kg/m^2^; and obesity: BMI of ≥30 kg/m2 [28]
Abdominal obesity	Waist‐to‐hip ratio (WHR) ≥ 0.90 for men and ≥ 0.85 for women [29]
Blood pressure (BP) status	High BP (hypertension): When the average systolic BP measurements are ≥140 mmHg or/and diastolic BP ≥90 mmHg, or/and hypertension diagnosis by healthcare providers and are on antihypertensive drugs; elevated BP (prehypertension): When the average systolic BP is ≥120 mmHg or/and diastolic BP ≥80 mmHg; and normal when the systolic BP is <120 mmHg and diastolic BP <80 mmHg [30]
Ten‐year cardiovascular risk	Low (<5% risk), mild (5% to <10%), moderate (10% to <20%), and high (≥20%) [31]
Knowledge	Knowledge was assessed using knowledge questions specific to each lifestyle risk factor and interpreted independently
Occupational status	Securely employed (government or private organization employee); insecure jobs (daily, factory, building, garment, garage driver, agriculture, and house duties), and unemployed (jobless, retired, and students)
Income	Very low income (less than 2250 Ethiopian birrs/month), low to middle (2251–5000), and middle (5001 and above)
Healthcare affordability	Healthcare affordability was assessed by participants’ “yes” or “no” responses to the question, “Can you afford to see a healthcare provider?”

Abbreviation: BMI, body mass index.

### Data analysis

Data were entered into Epi‐data software version 3.1 (the Epi Data Association, Denmark) and exported to Statistical Package for Social Sciences (SPSS, IBM Corp, USA) software version 28 for analysis. Pearson's chi‐square or Fisher's exact tests for categorical variables and one‐way analysis of variance (ANOVA) for continuous variables were used to examine risk factor differences between male and female participants. The tables and figures are used to summarize NCD risk factors, clusters of the risk factors, and participants’ knowledge about the risk factors. Clustering of risk factors was performed based on the number of NCD risk factors for each participant: less than three (0, 1, or 2) or at least three (3 and above) [32].

We used logistic regression analysis to investigate socioeconomic and demographic predictors of NCD risk factors and cardiovascular risk. The 10‐year CVD risk was dichotomized into two groups: low versus mild–moderate risks. In the bivariate analysis, we identified variables that had associations with the outcomes of interest at *p* < 0.30. We adjusted for these variables in the multivariable logistic regression analysis to identify significant predictors of 10‐year CVD risk. We examined the presence of collinearity among the variables included in the models; a variance inflation factor of <3 was achieved for all variables, indicating the absence of collinearity. The final models were also checked for nonsignificant Hosmer–Lemeshow goodness fit test (*p* > 0.05). The strength of association is presented using adjusted odds ratios (ORs) and 95% confidence intervals (CIs). The significance of association is declared at *p* < 0.05.

### Ethical considerations

This study received ethics approval from the Australian National University (ANU) Human Research Ethics Committee (Protocol number 2020/558), and local approval was obtained from Amhara Public Health Institute Research Ethics Review Committee, Bahir Dar, Ethiopia (approval number 1/10523).

## RESULTS

Of the 423 visited households that had eligible adults residing, 417 participated in this study. Table 2 summarizes the sociodemographic and healthcare access characteristics of the study participants. The mean age of the participants was 35.6 ± 12.6 years, and the median income was 6000 Ethiopian birr with an interquartile range of 6000, with 14% having a very low monthly income. More than half (57.3%) of the study participants have access to healthcare facilities within a 1‐km radius, whereas 36.2% could not afford medical expenses.

**TABLE 2:** Sociodemographic characteristics of adults for noncommunicable disease (NCD) risk factors study in Bahir Dar, Northwest Ethiopia, 2021 (*n* = 417).

Characteristics	Total, *n* (%)	Male, *n* (%)	Female, *n* (%)	*p* Value
**Age (years)**				
18–30	189 (45.3)	83 (43.9)	106 (56.1)	0.34
30–45	146 (35.0)	63 (43.2)	83 (56.8)	
46–65	82 (19.7)	43 (52.4)	39 (47.6)	
**Religion**				
Orthodox Christian	352 (84.4)	166 (47.2)	186 (52.8)	0.04
Muslim	51 (12.2)	15 (29.4)	36 (70.6)	
Others^a^	14 (3.4)	8 (57.1)	6 (42.9)	
**Educational status**				
Primary or lower	94 (22.5)	31 (33.0)	63 (67.0)	<0.01
Some/complete secondary	96 (23.0)	35 (36.5)	61 (63.5)	
Postsecondary (certificate or diploma)	73 (17.5)	27 (37.0)	46 (63.0)	
Degree and above	154 (36.9)	96 (62.3)	58 (37.7)	
**Occupation**				
Secure employment	137 (22.8)	84 (61.3)	53 (38.7)	
Insecure jobs	159 (39.1)	50 (31.4)	109 (68.6)	
Unemployed/retired/student	121 (29.0)	55 (45.5)	66 (54.5)	
**Marital status**				
Single	135 (32.4)	79 (58.5)	56 (41.5)	<0.01
Married	235 (56.4)	101 (43.0)	134 (57.0)	
Others^b^	47 (11.3)	9 (19.1)	38 (80.9)	
**Partner education (*n* = 237)**				
Primary or lower	55 (23.2)	28 (50.9)	27(49.1)	0.05
Some/complete secondary	57 (24.1)	26 (45.6)	31 (54.4)	
Postsecondary (certificate or diploma)	34 (14.3)	21 (61.8)	13 (38.2)	
Degree and above	91 (38.4)	27 (29.7)	64 (70.3)	
**Partner occupation (*n* = 237)**				
Secure employment	116 (48.9)	43 (37.1)	73 (62.9)	0.17
Insecure jobs^c^	105 (44.3)	52 (49.5)	53 (50.5)	
Unemployed/retired/student	16 (6.8)	7 (43.8)	9 (56.3)	
**Family size**				
1	35 (8.4)	22 (62.9)	13 (37.1)	0.08
2–4	205 (49.2)	92 (44.9)	113 (55.1)	
5+	177 (42.4)	75 (42.4)	102 (57.6)	
**Average monthly income (Ethiopian birr)** ^d^ **(*n* = 387)**				
00–2250	53 (13.7)	31 (58.5)	22 (41.5)	0.15
2251–5000	126 (32.6)	62 (49.2)	64 (50.8)	
>5000	208 (53.7)	88 (42.3)	120 (57.7)	
**Information access**				
Mobile phone	412 (98.8)	187(45.4)	225 (54.6)	1.00
Radio/television /computer	357 (85.6)	155 (43.4)	202 (56.6)	0.05
**Transport**				
Private car/motorcycle/ automobile	59 (14.1)	26 (44.1)	33 (55.9)	0.83
Bicycle	115 (27.6)	68 (59.1)	47 (40.9)	0.00
**Health check‐up history**				
Within the last 6 months	215 (51.6)	93 (43.3)	122 (56.7)	0.38
More than 6 months	202 (48.4)	96 (47.5)	106 (52.5)	
**Distance to a health facility**				
Within 1 km	239 (57.3)	103 (43.1)	136 (56.9)	0.29
More than 1 km	178 (42.7)	86 (48.3)	92 (51.7)	
**Healthcare affordability**	266 (63.8)	123 (46.2)	143 (53.8)	0.62
**Health insurance**	90 (21.6)	40 (44.4)	50 (55.6)	0.85

^a^ Protestant, Catholic, and other religions mentioned by the participants.

^b^ Living together but not married, separated, divorced, and widowed.

^c^ Daily work, factory, building, garment, garage, agriculture, drivers, house duties, and merchants.

^d^ One US Dollar is equivalent to 55.2 Ethiopian birrs.

### Prevalence of NCD risk factors, knowledge, and practices

Table 3 summarizes NCD risk factors, knowledge, and practices among adults in Bahir Dar.

**TABLE 3:** Prevalence of noncommunicable disease (NCD) risk factors, knowledge, and practices among adults in Bahir Dar, Northwest Ethiopia, 2021 (*n* = 417).

Variable category	Total, *n* (%)	Male, *n* (%)	Female, *n* (%)	*p* Value
**Dietary habits**				
Inadequate fruits and vegetable intake	399 (95.7)	184 (46.1)	215 (53.9)	0.12
Food habit of consumption of additional salt with food (7 days/week)	261 (62.6)	119 (45.6)	142 (54.4)	0.88
Soft drink intake (≥3 days/week)	68 (16.3)	35 (51.5)	33 (48.5)	0.26
Preference for fatty food	257 (61.6)	122 (47.5)	135 (52.5)	0.26
Purchased food intake (>5 days/week)	30 (7.2)	22 (73.3)	8 (26.7)	<0.01
**Physical activity**				
Inadequate physical activity	158 (37.9)	46 (29.1)	112 (70.9)	<0.01
Knowledge of prevention of NCDs with physical activity	317 (76.9)	146 (46.1)	171 (53.9)	0.62
**Alcohol consumption**				
History of alcohol use	330 (79.1)	170 (51.5)	160 (48.5)	<0.01
Alcohol use in the past 12 months	268 (64.2)	141 (52.6)	127 (47.4)	0.40
Current alcohol use	221 (53.0)	119 (53.8)	102 (46.2)	0.38
Heavy alcohol use (≥5 alcoholic drinks per occasion)	21 (5.1)	18 (85.7)	3 (14.3)	0.04
Knowledge about the harmful effect of alcohol	412 (98.8)	186 (45.0)	226 (55.0)	0.39
**Tobacco use**				
History of tobacco use	25 (6.0)	21 (84.0)	4 (16.0)	<0.01
Former tobacco user	17 (4.1)	15 (88.2)	2 (11.8)	0.57
Current tobacco users	8 (1.9)	6 (75.0)	2 (25.0)	0.54
History of smokeless tobacco use	2 (0.5)	2 (100)	0 (0)	–
Exposure to home smoke	16 (3.8)	5 (31.3)	11 (68.8)	0.31
Exposure to workplace smoke	18 (4.3)	8 (44.4)	10 (55.6)	0.06
Knowledge about the danger of tobacco smoke	403 (96.6)	184 (45.7)	219 (54.3)	0.54
**Khat chewing**				
History of khat chewing	42 (10.1)	27 (64.3)	15 (35.7)	0.01
Former khat chewer	21 (5.1)	16 (76.2)	5 (23.8)	0.10
Current khat chewer	21 (5.1)	11 (52.4)	10 (47.6)	0.50
**Waist‐to‐hip ratio values (*n* = 408)**				
Abdominal obese	275 (67.4)	104 (37.8)	171 (62.2)	<0.01
Normal	133 (32.6)	80 (60.2)	53 (39.8)	
**Body mass index values (*n* = 412)**				
Underweight	23 (5.6)	16 (69.6)	7 (30.4)	<0.01
Normal weight	267 (64.8)	126 (47.2)	141 (52.8)	
Overweight	87 (21.1)	37 (42.5)	50 (57.5)	
Obese	35 (8.5)	9 (25.7)	26 (74.3)	
**Blood pressure status (*n* = 415)**				
Normal^a^	282 (68.0)	121(42.9)	161(57.1)	0.23
Elevated blood pressure^b^	47 (11.3)	26 (55.3)	21 (44.7)	
High blood pressure^c^	86 (20.7)	41 (47.7)	45 (52.3)	

^a^ Normal blood pressure (BP): when the systolic BP is <120 mmHg and diastolic BP <80 mmHg.

^b^ Elevated blood pressure: when the average systolic BP is ≥120 mmHg or/and diastolic BP ≥80 mmHg.

^c^ High blood pressure: when the average systolic BP are ≥140 mmHg or/and diastolic BP ≥90 mmHg or/and hypertension diagnosis by healthcare providers and are on antihypertensive drugs.

**Dietary habits**: The majority (95.7%) of participants in this study have inadequate fruit and vegetable intake. The main reasons mentioned for not having an adequate intake of fruits and vegetables were of high price (67.3%), culture or diet customs (26.7%), and not being available in the nearby market (19.5%). High salt intake was reported by 87.8% of participants, soft drink intake by 16.3%, and purchased food consumption by 7.2%.

**Physical activity**: Over one third (37.9%) of adults have insufficient physical activity levels. Nevertheless, most (76.9%) of adults know that adequate physical activity prevents NCDs. The main reasons for not doing physical activities were lack of interest (68.1%), time constraints (37.4%), and not knowing how to do (31.4%).

**Alcohol consumption**: Most (92.6%) participants know alcohol harms health; however, 53% are current alcohol consumers, and 12.2% are heavy drinkers, which is more common in men than women (*p* < 0.01). The main reasons given for alcohol consumption are celebrations (57.1%), drinking with friends (45.5%), and relaxing/enjoying (38.1%).

**Tobacco and *khat* use**: Overall, 6% of participants have a history of tobacco use, whereas 1.9% are current tobacco users, and 5.0% are current *khat* chewers. The main reasons mentioned for tobacco use are peer pressure (71.9%), stress (56.0%), curiosity (41.6%), and modernity (40.4%).

**Overweight and obesity**: According to BMI categories, 29.6% of participants were overweight or obese: 24.5% in males and 33.9% in females. Using waist‐to‐hip (WHR) ratio, 67.2% of participants were abdominally obese, 56.5% of males, and 76.3% of females (*p* < 0.05).

**Hypertension**: The prevalence of hypertension in this study is 20.7%. Of hypertensive participants, 32.6% were undiagnosed, 41.8% had a history of hypertension, and 25.6% were on antihypertensive drugs.

**FIGURE 1:**
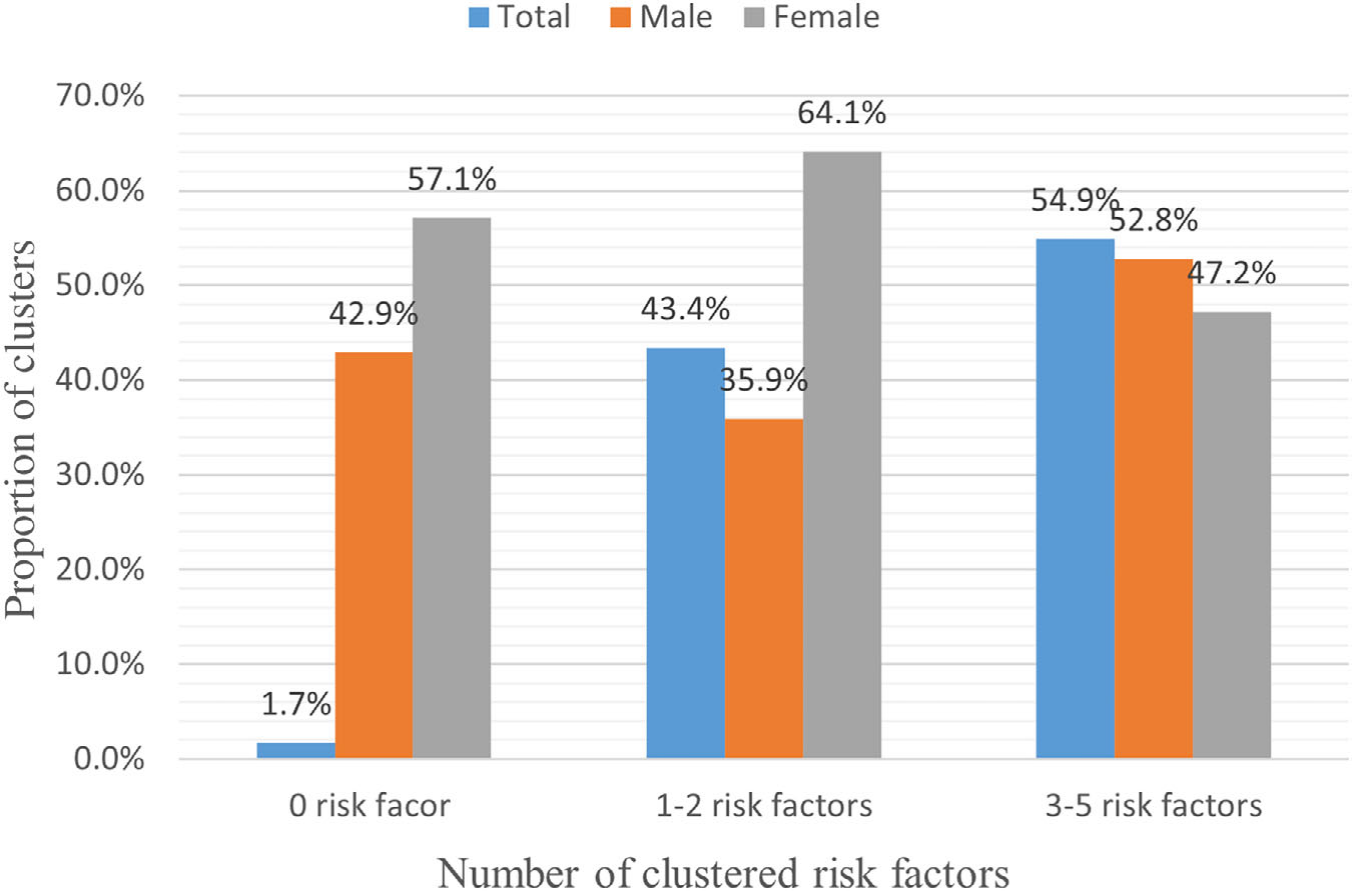
Prevalence of clustered risk factors for noncommunicable diseases (NCDs) among adults in Bahir Dar, Northwest Ethiopia, 2021.

**Clustering of NCD risk factors**: More than half (54.9%) of participants in this study have clusters of three or more NCD risk factors, 43.4% have 1–2 risk factors, and only 1.7% were free from risk factors. Clusters of three or more risk factors are higher in males than females (*p* < 0.01) (Figure 1).

Table 4 summarizes the association between NCD risk factors and the socioeconomic and demographic variables.

**TABLE 4:** Associations (adjusted odd ratio [OR] and 95% confidence interval [CI]) between socioeconomic and demographic variables and noncommunicable disease (NCD) risk factors among adults in Bahir Dar, Northwest Ethiopia, 2021.

Variables	Overweight/obesity	Abdominal obesity	Hypertension	Fruits/vegetable intake	Physical inactivity	Alcohol use	Khat use
	Adjusted OR (95% CI)	Adjusted OR (95% CI)	Adjusted OR (95%CI)	Adjusted OR (95% CI)	Adjusted OR (95% CI)	Adjusted OR (95% CI)	Adjusted OR (95% CI)
**Sex**							
Males	0.9 (0.5, 1.4)	0.4 (0.2, 0.7)*	NA	1.2 (0.4, 4.2)	0.4 (0.3, 0.7)*	1.9 (1.2, 3.0)*	NA
Females	Ref						
**Age (years)**							
18–30	0.7 (0.3, 1.5)	0.2 (0.1, 0.6)*	0.1 (0.1, 0.2)*	0.2 (0.1, 1.3)	1.1 (0.5, 2.4)	1.3 (0.6, 2.9)	0.2 (0.1, 1.0)
31–44	1.2 (0.6, 2.3)	0.3 (0.1, 0.8)*	0.2 (0.1, 0.3)*	0.5 (0.1, 2.5)	1.2 (0.6, 2.4)	2.2 (1.1, 4.5)*	0.7 (0.2, 2.3)
46–65	Ref						
**Religion**							
Christian	1.1 (0.6, 2.0)	1.1 (0.5, 2.1)	1.2 (0.6, 2.4)	3.3 (1.1, 10.6)*	0.9 (0.5, 1.7)	11.1 (4.8, 25)*	0.1 (0.1, 0.3)*
Muslim^a^	Ref						
**Marital status**							
Married	3.2 (1.6, 6.4)*	2.5 (1.4, 4.6)*	1.4 (0.6, 3.3)	0.2 (0.1,1.1)	1.8 (0.9, 3.3)	1.0 (0.6, 1.9)	0.3 (0.1, 1.3)
Others^b^	6.3 (2.3, 16.8)*	2.7 (0.9, 8.0)*	2.5 (0.8, 7.4)	0.6 (0.1,10.0)	3.5 (1.3, 9.0)*	0.6 (0.2,1.5)	0.3 (0.1, 2.1)
Single	Ref						
**Education status**							
Low/primary	0.9 (0.5, 1.8)	1.0 (0.5, 2.1)	1.0 (0.5, 2.0)	NA	1.2 (0.6, 2.4)	1.0 (0.5,1.9)	1.8 (0.4, 7.6)
Some/secondary^c^	0.8 (0.4, 1.5)	0.8 (0.5, 1.5)	0.7 (0.3, 1.4)	NA	0.9 (0.5, 1.6)	0.8 (0.5,1.4)	1.2 (0.2, 5.5)
College above	Ref						
**Occupation**							
Securely employed	0.6 (0.3, 1.2)	0.7 (0.4, 1.4)	NA	5.2 (1.1, 26.7)*	0.6 (0.3, 1.1)	1.5 (0.9, 2.7)	0.9 (0.2, 2.6)
Unemployed^d^	1.0 (0.5, 1.8)	0.6 (0.3, 1.2)	NA	1.1 (0.3, 4.1)	0.9 (0.5, 1.6)	1.4 (0.8,2.6)	0.7(0.2, 2.9)
Insecure jobs^e^	Ref						
**Family size (individuals)**							
1–2	0.7 (0.3, 1.6)	1.0 (0.5, 2.1)	NA	NA	NA	1.0 (0.5, 1.9)	NA
3–4	1.0 (0.6, 1.6)	1.5 (0.8, 2.6)	NA	NA	NA	1.5 (0.9, 2.5)	NA
5+	Ref						
**Average monthly income (Ethiopian birr)** ^f^							
<2250	NA	NA	NA	1.1 (0.3, 4.7)	1.5 (0.8, 3.2)	NA	NA
2251–5000	NA	NA	NA	4.7 (1.0, 22.6)	1.9 (1.1, 3.1)*	NA	NA
>5000	Ref						

Abbreviations: Adjusted odds ratio, adjusted OR; confidence interval, CI; NA, not applicable (when the cells contain less than five observations); NA, not adjusted; Ref: reference.

^*^ Significant association *p* < 0.05.

^a^ Muslim, protestant, and other religions.

^b^ Living together but not married, separated, divorced, and widowed.

^c^ Secondary school education (grades 9–12).

^d^ Daily work, factory, building, garment, garage, agriculture, driver, house duties, and merchant.

^e^ Unemployed, retired, and student.

^f^ One US dollar is 54.2 Ethiopian birrs.

Married participants are 3.2 times more likely to be overweight/obese (adjusted OR 3.2, 95% CI 1.6–6.4) and two times more likely to have abdominal obesity than single participants (adjusted OR 2.5, 95% CI 1.4–4.6). Males are 60% less likely to have abdominal obesity than female participants (adjusted OR 0.4, 95% CI 0.2–0.7), and older adults are 80% more likely to have abdominal obesity than young adults (adjusted OR 0.2, 95% CI 0.1–0.6).

Adults between the ages of 45 and 65 are significantly more likely to have hypertension than young adults (adjusted OR 0.1, 95% CI 0.1–0.6) and 80% more likely to have hypertension than adults between the ages of 31 and 44 (adjusted OR 0.2, 95% CI 0.1–0.3).

Participants who are securely employed are 5.2 times more likely to have adequate fruit and vegetable intake than participants with insecure jobs (adjusted OR 5.2, 95% CI 1.2–26.7), and Christians are 3.3 times more likely to have adequate fruit and vegetable intake than others (adjusted OR 3.3, 95% CI 1.1–10.6).

Insufficient physical activity is significantly more common in women than men (adjusted OR 0.4, 95% CI 0.3–0.7) and low‐income participants (adjusted OR 1.9, 95% CI 1.1–3.1). With regard to alcohol, adults who are between the ages of 31 and 45 (adjusted OR 2.2, 95% CI 1.1–4.5), males (adjusted OR 1.9, 95% CI 1.2–3.0), and Christians (adjusted OR 11.1, 95% CI 4.8–25) are more likely to consume alcohol than their counterparts. *Khat* chewing is significantly lower in Christians than in other religions, such as Muslims (adjusted OR 0.1, 95% CI 0.1–0.3).

**NB**: the results are obtained after adjusted for socioeconomic (education, occupation, and income) and demographic variables (age, sex, religion, marital status, and family size); tobacco use is not adjusted for socioeconomic variables due to the small number of observations (less than five cases in a category); and high salt intake does not show significant association with the socioeconomic and demographic variables.

### Cardiovascular disease risk stratification

**FIGURE 2:**
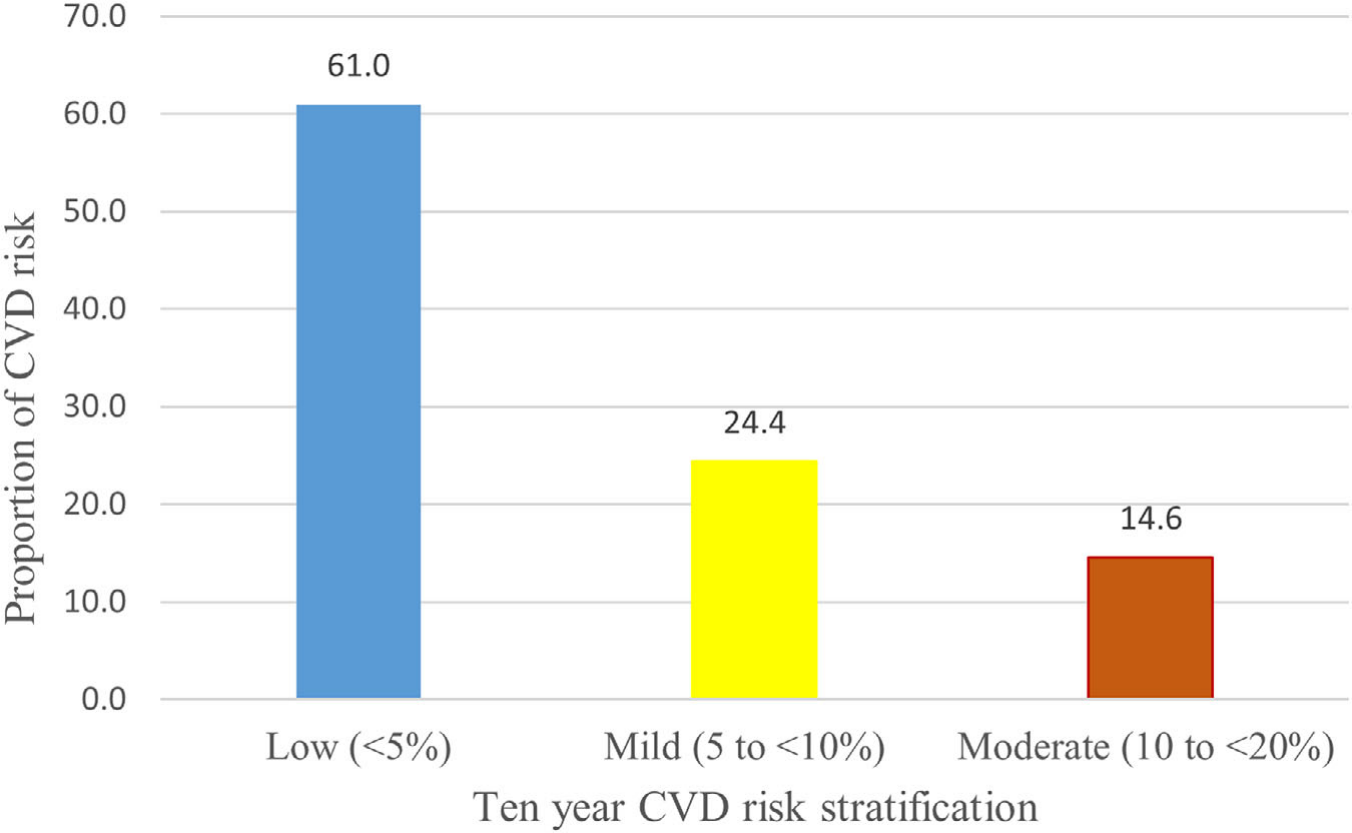
A 10‐year cardiovascular disease (CVD) risk stratification among adults in Bahir Dar, Northwest Ethiopia, 2021.

Using CVD risk algorithm, 61.0% of the participating adults above 40 have a 10‐year CVD risk of less than 5%, 24.4% have risks between 5% and 10%, and 14.6% have risks between 10% and 20% (Figure 2).

### Predictors of 10‐year CVD risk among adults

The final predictors of 10‐year CVD risk are sex, WHR, and clustered risk factors. Male participants are four times more likely to have a moderate (10%–20%) 10‐year CVD risk than females (adjusted OR 4.2, 95% CI 1.5–11.4). Participants with abdominal obesity are six times more likely to have a 10%–20% 10‐year CVD risk than adults with a normal WHR (adjusted OR 5.8, 95% CI 1.4–24.2). Participants with clusters of three or more risk factors are four times more likely to have 10%–20% CVD risk than participants without clusters of the risk factors (adjusted OR 4.0, 95% CI 1.6–10.3) (Table 5).

**TABLE 5:** Predictors of 10‐year risk for cardiovascular disease (CVD) amongst participants among adults in Bahir Dar, Northwest Ethiopia, 2021.

Variables	Cardiovascular risk (‐mild‐to‐moderate vs. low)	95% Confidence intervals		
	Adjusted odds ratio	Lower limit	Upper limit	*p* Value
**Sex**				
Males	4.15	1.51	11.40	<0.01
Females	1			
**Marital status**				
Single	0.36	0.12	1.08	0.07
Married				
**Educational status**				
Primary or lower	0.69	0.22	2.19	0.52
Some/secondary	0.29	0.07	1.14	0.08
Postsecondary	1			
**Occupation**				
Employed	0.47	0.15	1.49	0.20
Unemployed	1			
**Waist‐hip ratio (abnormal)**	5.80	1.39	24.20	0.01
**Alcohol use**	2.07	0.84	5.10	0.11
**Clustered risk factors**	4.01	1.56	10.33	<0.01

*Note*: Significant association: *p* < 0.05.

## DISCUSSION

This study shows that adults in Bahir Dar are at a high risk of prematurely developing NCDs, with more than half having clusters of three or more risk factors and one in six adults above 40 having a moderate level of 10‐year cardiovascular risk.

Despite tobacco use being rare, other risk factors are higher amongst participants in this study than in the 2015 national survey [16], a study of employees in Mekelle [17], and a survey in Southwest Ethiopia [18]. The difference could be due to the passage of time and study area variations among the surveys. The risk factors prevalence is also higher than reports from South Africa [33], India [34], China [35], and the WHO global report (23% physical inactivity, 38% alcohol use, and 22% tobacco use) [36]. Differences might be due to variations in participants’ sociodemographic and economic characteristics. Higher poverty, unemployment, and low educational status could contribute to a high prevalence of risk factors in the study area. In addition, the current political chaos in the country [37], the global COVID‐19 pandemic during the study period [38], poor healthcare access, and low levels of screening and treatment for NCDs potentially raise the prevalence of the risk factors.

This study found a higher prevalence of inadequate fruit and vegetable intake than a study in Southwest Ethiopia (27%) [18], but slightly lower than the national report (98.7%) [16] and a study (99%) in Northern Ethiopia [17]. The differences could be due to variations in climatic zones limiting the agricultural products and their availability in the market and the prevailing food cultures/taboos. The study shows that securely employed participants consume more fruits and vegetables than participants with insecure jobs. Employed adults might have had more exposure to health information in their workplace, from colleagues, and the cumulative knowledge leading to seeking good health and prevention of diseases [39]. The ability to purchase fruits and vegetables might be better in older and employed adults than in younger or unemployed groups.

The level of physical inactivity in this study is higher than the global prevalence rate (23%) [40], and a report (20%) based on 22 African countries [41]; however, higher rates were reported in other African countries, such as Uganda (51%) [42], and Somaliland (78%) [43]. The main reasons for insufficient physical activity were a lack of interest and time to exercise. Women participants are more physically inactive than men, which aligns with a study finding that individual‐level factors such as sex, self‐efficacy, and motivation significantly correlate with physical activity levels [44].

This study finds a higher rate of alcohol consumption than a 40.7% national report [16], and from a 9.3% prevalence in Southwest Ethiopia [18]. Socioeconomic and cultural variations may account for the differences among the study areas. Consistent with other studies, male participants are higher to consume alcohol than women [45]. In Ethiopia, men are mostly family breadwinners, work outside the home for long hours, and are exposed to stressful conditions, and cultural acceptance of men drinking might lead to higher alcohol consumption in men.

The prevalence of current tobacco use in this study is lower than that found in systematic review (6.7%) in Ethiopia [46], prevalence (9%) in school‐going students in East Africa [47], and from the 2021 WHO report (22%) [48].

The prevalence of overweight or obesity in this study is consistent with reports in Gonder (32%) [49], and Mekelle Northern region (30%) [17], and close to worldwide prevalence (39%) [28]. Marital status is a significant predictor for the prevalence of overweight/obesity. This could be because when people get married, their level of engagement in physical activities decreases, they give less attention to their body size/shape, and they increase consumption of fatty foods.

Hypertension prevalence in this population is higher than the 16% national prevalence [50], but lower than the 27% prevalence in Gonder [51], and 35% in Addis Ababa [52]. The significant sociodemographic predictor for hypertension prevalence is the age of participants. This factor could explain hypertension in various ways and inform targeted interventions to reduce its burden.

Additionally, this study shows that one in six participants have a moderate level (10%–20%) of 10‐year CVD risk. High CVD risk (>20%) was not observed in this study population. However, the moderate risk category is higher than reports from other countries such as Nigeria (76.9%, 8.5%, and 14.6%) [53], Angola (87.6%, 7.9%, and 4.5%) [54], Mozambique (90.2%, 6.7%, and 3.3%) [55], India (83%, 6.8%, and 10.2%) [56], and in Vietnam (89.2%, 5.1%, and 5.1%) [57] reported from the low, moderate, and high‐risk category, respectively. Country‐specific variations such as socioeconomic differences, access to preventive health services, and health literacy levels might contribute to this variation.

This study reveals that male adults have a higher CVD risk than females, consistent with findings in Finland [58], and Vietnam [57]. Also, in some developed states like England, CVD risk indicators were significantly higher in men [59]. In the current study, although physical inactivity and abdominal obesity levels are higher in females, clusters of three or more risk factors are higher in males than females, which increases the overall CVD risk levels.

Abdominal obesity is a significant predictor for CVD risk level, similar to the Nigerian study that reported that people who have increased waist circumference are three times higher to have CVD risk than normal participants [53], and in Londrina Medical Center women having abdominal obesity were two times higher to have CVD risk than normal women [60]. A study in China also shows that abdominal obesity is strongly associated with CVD diseases [61].

The current study reveals that adults with clusters of three or more risk factors are significantly associated with higher CVD risk. This finding is consistent with a study in Brazil that showed that clustered risk factors such as obesity, low fruit/vegetable intake, and lack of vigorous or moderate‐intensity activities are significantly associated with the risk of CVD diseases [62]. Multiple combined risks are demonstrated in other studies to increase CVD risks [63].

### Study limitations

This study provides relevant information on the prevalence of NCD risk factors among adults in Bahir Dar. Nevertheless, the study may have the following limitations: (1) The data were collected through interviews, which might have suffered some social desirability bias related to some of some risk factors such as tobacco use; (2) the study did not incorporate biological samples to determine metabolic risks; thus, the interpretation of results is limited to interviews and physical measurements, and (3) finally, this study was limited to a particular area of Ethiopia with relatively small sample size, the findings, therefore, could not represent the whole adults in the country.

## CONCLUSION

This study adds to the limited evidence available to inform actions to reduce rising rates of NCDs, a current public health issue in Ethiopia. The results show that more than half of adults in Bahir Dar have clusters of three or more risk factors, and one in six adults above 40 have a moderate level of 10‐year CVD risk. The prevalence of the risk factors varies significantly across adults’ socioeconomic and demographic characteristics. The CVD risk is high among males, those with abdominal obesity, and clustered risk factors.

## AUTHOR CONTRIBUTIONS

Tilahun Tewabe Alamnia , Ginny M. Sargent, and Matthew Kelly conceived this study. All authors participated in the analysis, interpretation, drafting, and critically reviewing the manuscript for intellectual content and developed the final version for submission. All authors read and approved this manuscript.

## CONFLICT OF INTEREST STATEMENT

All authors declare no conflicts of interest.

## ETHICS STATEMENT

The Australian National University (ANU) Human Research Ethics Committee (protocol number 2020/558) and Amhara Public Health Institute (APHI) Research Ethics Review Committee, Bahir Dar, Ethiopia (protocol number 1/10523) approved this study. All participants provided informed consent to be eligible to participate in this study.

## Data Availability

All data relevant to this study are included in the manuscript.

## References

[1] World Health Organization . Noncommunicable Diseases. Key Fact s. World Health Organization; 2022. https://www.who.int/news‐room/fact‐sheets/detail/noncommunicable‐diseases

[2] Frumkin H , Haines A . Global environmental change and noncommunicable disease risks. Annu Rev Public Health. 2019;40:261‐282. 30633714 10.1146/annurev-publhealth-040218-043706

[3] Popkin BM . Technology, transport, globalization and the nutrition transition food policy. Food Policy. 2006;31(6):554‐569.

[4] Gaskin CJ , Orellana L . Factors associated with physical activity and sedentary behavior in older adults from six low‐and middle‐income countries. Int J Environ Res Public Health. 2018;15(5):908. 29751561 10.3390/ijerph15050908 PMC5981947

[5] Beaglehole R , Bonita R , Horton R , et al. Priority actions for the non‐communicable disease crisis. Lancet North Am Ed. 2011;377(9775):1438‐1447. 10.1016/S0140-6736(11)60393-0 21474174

[6] Bennett JE , Stevens GA , Mathers CD , et al. NCD countdown 2030: worldwide trends in non‐communicable disease mortality and progress towards Sustainable Development Goal target 3.4. Lancet North Am Ed. 2018;392(10152):1072‐1088. 10.1016/S0140-6736(18)31992-5 30264707

[7] World Health Organization . Global Action Plan for the Prevention and Control of Noncommunicable Diseases 2013–2020 . World Health Organization; 2013.

[8] Ministry of Health Ethiopia . Health Sector Transformation Plan II (2020/21‐2024/25). Ministry of Health Ethiopia, Addis Ababa, Ethiopia; 2021.

[9] Tafirenyika M . The changing face of Ethiopia. Africa Renewal. 2015. https://www.un.org/africarenewal/magazine/august‐2015/changing‐face‐ethiopia

[10] Misganaw A , Haregu TN , Deribe K , et al. National mortality burden due to communicable, non‐communicable, and other diseases in Ethiopia, 1990–2015: findings from the Global Burden of Disease Study 2015. Popul. Health Metr. 2017;15(1):29. 28736507 10.1186/s12963-017-0145-1 PMC5521057

[11] Harlan JR . Ethiopia: a center of diversity. Econ Bot. 1969;23(4):309‐314.

[12] Seifu Y , Habte M , Alayu S . The demographic transition and development nexus in Ethiopia: real dividend or burden? In: The Demographic Transition and Development in Africa. Springer; 2011:69‐88.

[13] World Health Organization . Global Health Estimates 2019 . WHO; 2020.

[14] Federal Democratic Republic of Ethiopia Ministry of Health . Guidelines on Clinical and Programmatic Management of Major Non Communicable Diseases . FMOH, Addis Ababa; 2016.

[15] Shiferaw F , Letebo M , Misganaw A , et al. Non‐communicable diseases in Ethiopia: disease burden, gaps in health care delivery and strategic directions. Ethiop J Health Dev. 2018;32(3):1‐11.

[16] Ethiopian Federal Ministry of Health. Ethiopia Public Health Institute . World Health Organization: Ethiopia STEPS Report on Risk Factors for Chronic Noncommunicable Diseases and Prevalence of Selected NCDs . Ethiopian Public Health Institute; 2016.

[17] Gebremariam LW , Chiang C , Yatsuya H , et al. Non‐communicable disease risk factor profile among public employees in a regional city in northern Ethiopia. Sci Rep. 2018;8(1):1‐11. 29915239 10.1038/s41598-018-27519-6 PMC6006379

[18] Alemseged F , Haileamlak A , Tegegn A , et al. Risk factors for chronic non‐communicable diseases at Gilgel Gibe Field Research Center, Southwest Ethiopia: population based study. Ethiop J Health Sci. 2012;22(4):19‐28. PMC3542740 23319837

[19] STROBE . STROBE Statement—Checklist of Items that Should Be Included in Reports of Cross‐Sectional Studies . STROBE. 2007. https://www.equator‐network.org/wp‐content/uploads/2015/10/STROBE_checklist_v4_cross‐sectional.pdf 10.1007/s00038-007-0239-9 18522360

[20] World Health Organization . WHO STEPS Instrument (Core and Expanded). WHO; 2015. http://www/who/int/chp/steps/STEPS_Instrument_v2

[21] Demaio AR , Dugee O , Amgalan G , et al. Protocol for a national, mixed‐methods knowledge, attitudes and practices survey on non‐communicable diseases. BMC Public Health. 2011;11(1):961. 22208645 10.1186/1471-2458-11-961 PMC3280340

[22] Salwa M , Haque MA , Khalequzzaman M , Al Mamun MA , Bhuiyan MR , Choudhury SR . Towards reducing behavioral risk factors of non‐communicable diseases among adolescents: protocol for a school‐based health education program in Bangladesh. BMC Public Health. 2019;19(1):1002. 31345186 10.1186/s12889-019-7229-8 PMC6659286

[23] Gelibo T , Amenu K , Taddele T , et al. Low fruit and vegetable intake and its associated factors in Ethiopia: a community based cross sectional NCD steps survey. Ethiop J Health Dev. 2017;31(1):355‐361.

[24] World Health Organization . Global Recommendations on Physical Activity for Health. WHO; 2011. 26180873

[25] Alebachew W , Semahegn A , Ali T , Mekonnen H . Prevalence, associated factors and consequences of substance use among health and medical science students of Haramaya University, eastern Ethiopia, 2018: a cross‐sectional study. BMC Psychiatry. 2019;19(1):343. 31694591 10.1186/s12888-019-2340-z PMC6836499

[26] Reda AA , Kotz D , Biadgilign S . Adult tobacco use practice and its correlates in eastern Ethiopia: a cross‐sectional study. Harm Reduct J. 2013;10(1):28. 24171800 10.1186/1477-7517-10-28 PMC4175506

[27] Gizaw AT , Amdisa D , Lemu YK . Predictors of substance use among Jimma University instructors, Southwest Ethiopia. Subst Abuse Treat Prevent Policy. 2020;15(1):2. 10.1186/s13011-019-0248-8 PMC6950981 31915036

[28] World Health Organisation . Obesity and Overweight . WHO; 2021. https://www.who.int/news‐room/fact‐sheets/detail/obesity‐and‐overweight

[29] World Health Organisation . Definition, Diagnosis and Classification of Diabetes Mellitus and Its Complications (Report of a WHO consultation 1999). WHO; 1999.

[30] World Health Organization . A Global Brief on Hypertension: Silent Killer, Global Public Health Crisis: World Health Day 2013 . World Health Organization; 2013.

[31] World Health Organization . Hearts: Technical Package for Cardiovascular Disease Management in Primary Health Care . World Health Organization; 2020.

[32] Dahal S , Sah RB , Niraula SR , Karkee R , Chakravartty A . Prevalence and determinants of non‐communicable disease risk factors among adult population of Kathmandu. PLoS ONE. 2021;16(9):e0257037. 34495984 10.1371/journal.pone.0257037 PMC8425558

[33] Wandai M , Day C . Trends in Risk Factors for Non‐Communicable Diseases in South Africa . Health Systems Trust; 2015:27.

[34] Sarma P , Sadanandan R , Thulaseedharan JV , et al. Prevalence of risk factors of non‐communicable diseases in Kerala, India: results of a cross‐sectional study. BMJ Open. 2019;9(11):e027880. 10.1136/bmjopen-2018-027880 PMC6858196 31712329

[35] Li Y , Wang L , Jiang Y , Zhang M , Wang L . Risk factors for noncommunicable chronic diseases in women in China: surveillance efforts. Bull World Health Organ. 2013;91:650‐660. 24101781 10.2471/BLT.13.117549 PMC3790222

[36] World Health Organization . Noncommunicable Diseases (NCD) Country Profiles, 2018 [Online] . World Health Organization; 2018.

[37] Ethiopia Human Rights Watch . World Report 2021 . Ethiopia Human Rights Watch. https://www.hrw.org/world‐report/2021/country‐chapters/ethiopia

[38] Vindegaard N , Benros ME . COVID‐19 pandemic and mental health consequences: systematic review of the current evidence. Brain Behav Immun. 2020;89:531‐542. 32485289 10.1016/j.bbi.2020.05.048 PMC7260522

[39] Furuya Y , Kondo N , Yamagata Z , Hashimoto H . Health literacy, socioeconomic status and self‐rated health in Japan. Health Promot Int. 2015;30(3):505‐513. 24131729 10.1093/heapro/dat071

[40] Sallis JF , Bull F , Guthold R , et al. Progress in physical activity over the Olympic quadrennium. Lancet North Am Ed. 2016;388(10051):1325‐1336. 10.1016/S0140-6736(16)30581-5 27475270

[41] Guthold R , Louazani SA , Riley LM , et al. Physical activity in 22 African countries: results from the World Health Organization STEPwise approach to chronic disease risk factor surveillance. Am J Prev Med. 2011;41(1):52‐60. 21665063 10.1016/j.amepre.2011.03.008

[42] Mondo CK , Otim MA , Musoke R , Akol G , Orem J . The prevalence and distribution of non‐communicable diseases and their risk factors in Kasese district, Uganda. CardiovascJ Afr. 2013;24(3):52. 23736126 10.5830/CVJA-2012-081 PMC3721879

[43] Ahmed SH , Meyer HE , Kjøllesdal MK , et al. The prevalence of selected risk factors for non‐communicable diseases in Hargeisa, Somaliland: a cross‐sectional study. BMC Public Health. 2019;19(1):878. 31272414 10.1186/s12889-019-7101-x PMC6611144

[44] Bauman AE , Reis RS , Sallis JF , Wells JC, Loos RJ, Martin BW. Correlates of physical activity: why are some people physically active and others not? Lancet North Am Ed. 2012;380(9838):258‐271. 10.1016/S0140-6736(12)60735-1 22818938

[45] Malaju MT , Asale GA . Association of khat and alcohol use with HIV infection and age at first sexual initiation among youths visiting HIV testing and counseling centers in Gamo‐Gofa Zone, South West Ethiopia. BMC Int Health Human Rights. 2013;13(1):10. 10.1186/1472-698X-13-10 PMC3568407 23375131

[46] Ayano G , Solomon M , Hibdiye G , Duko B . The epidemiology of tobacco use in Ethiopia: a systematic review and meta‐analysis. J Public Health. 2020;30:1143‐1153.

[47] Tezera N , Endalamaw A . Current cigarette smoking and its predictors among school‐going adolescents in East Africa: a systematic review and meta‐analysis. Int J Pediatr. 2019;2019:4769820. 31205474 10.1155/2019/4769820 PMC6530160

[48] World Health Organization . WHO Global Report on Trends in Prevalence of Tobacco Use 2000–2025 . World Health Organization; 2021.

[49] Moges B , Amare B , Fantahun B , Kassu A . High prevalence of overweight, obesity, and hypertension with increased risk to cardiovascular disorders among adults in northwest Ethiopia: a cross sectional study. BMC Cardiovasc Disord. 2014;14(1):155. 25373922 10.1186/1471-2261-14-155 PMC4228065

[50] Gebreyes YF , Goshu DY , Geletew TK , et al. Prevalence of high bloodpressure, hyperglycemia, dyslipidemia, metabolic syndrome and their determinants in Ethiopia: evidences from the National NCDs STEPS Survey, 2015. PLoS One. 2018;13(5):e0194819. 29742131 10.1371/journal.pone.0194819 PMC5942803

[51] Demisse AG , Greffie ES , Abebe SM , et al. High burden of hypertension across the age groups among residents of Gondar city in Ethiopia: a population based cross sectional study. BMC Public Health. 2017;17(1):647. 28793889 10.1186/s12889-017-4646-4 PMC5551023

[52] Abebe S , Yallew WW . Prevalence of hypertension among adult outpatient clients in hospitals and its associated factors in Addis Ababa, Ethiopia: a hospital based cross‐sectional study. BMC Res Notes. 2019;12(1):87. 30764864 10.1186/s13104-019-4127-1 PMC6376725

[53] Babatunde OA , Olarewaju SO , Adeomi AA , et al. 10‐Year risk for cardiovascular diseases using WHO prediction chart: findings from the civil servants in South‐Western Nigeria. BMC Cardiovasc Disord. 2020;20(1):1‐10. 32234017 10.1186/s12872-020-01438-9 PMC7110661

[54] Pedro JM , Brito M , Barros H . Cardiovascular risk assessment in Angolan adults: a descriptive analysis from Cardiobengo, a community‐based survey. Int J Hypertens. 2018;2018:2532345. 30258655 10.1155/2018/2532345 PMC6146561

[55] Damasceno A , Padrão P , Silva‐Matos C , Prista A , Azevedo A , Lunet N . Cardiovascular risk in Mozambique: who should be treated for hypertension? J Hypertens. 2013;31(12):2348. 24220589 10.1097/HJH.0b013e3283656a0a PMC4164281

[56] Ghorpade AG , Shrivastava SR , Kar SS , Sarkar S , Majgi SM , Roy G . Estimation of the cardiovascular risk using World Health Organization/International Society of Hypertension (WHO/ISH) risk prediction charts in a rural population of South India. Int J Health Policy Manage. 2015;4(8):531. 10.15171/ijhpm.2015.88 PMC4529043 26340393

[57] Anh Hien H , Tam NM , Tam V , et al. Estimation of the cardiovascular risk using world health organization/international society of hypertension risk prediction charts in Central Vietnam. PLoS ONE. 2020;15(11):e0242666. 33227012 10.1371/journal.pone.0242666 PMC7682854

[58] Jousilahti P , Vartiainen E , Tuomilehto J , Puska P . Sex, age, cardiovascular risk factors, and coronary heart disease: a prospective follow‐up study of 14 786 middle‐aged men and women in Finland. Circulation. 1999;99(9):1165‐1172. 10069784 10.1161/01.cir.99.9.1165

[59] Pinho‐Gomes AC , Peters SA , Thomson B , Woodward M . Sex differences in prevalence, treatment and control of cardiovascular risk factors in England. Heart. 2021;107(6):462‐467. 10.1136/heartjnl-2020-317446 32887737

[60] Cabrera MA , Gebara OC , Diament J , Nussbacher A , Rosano G , Wajngarten M . Metabolic syndrome, abdominal obesity, and cardiovascular risk in elderly women. Int J Cardiol. 2007;114(2):224‐229. 16784787 10.1016/j.ijcard.2006.01.019

[61] Fan H , Li X , Zheng L , et al. Abdominal obesity is strongly associated with cardiovascular disease and its risk factors in elderly and very elderly community‐dwelling Chinese. Sci Rep. 2016;6(1):21521. 26882876 10.1038/srep21521 PMC4756331

[62] Fuchs SC , Moreira LB , Camey SA , Moreira MB , Fuchs FD . Clustering of risk factors for cardiovascular disease among women in Southern Brazil: a population‐based study. Cad Saude Publica. 2008;24:s285‐s293. 18670708 10.1590/s0102-311x2008001400013

[63] Tegegne TK , Islam SM , Maddison R . Effects of lifestyle risk behaviour clustering on cardiovascular disease among UK adults: latent class analysis with distal outcomes. Sci Rep. 2022;12(1):1‐8. 36253519 10.1038/s41598-022-22469-6 PMC9576714

